# Polymorphism of alpha-1-antitrypsin in hematological malignancies

**DOI:** 10.1590/S1415-47572009005000085

**Published:** 2009-12-01

**Authors:** Aleksandra Topic, Zorica Juranic, Svetislav Jelic, Ivana Golubicic Magazinovic

**Affiliations:** 1 Department of Medical Biochemistry, Faculty of Pharmacy, University of BelgradeSerbia; 2 Department of Experimental Oncology, Institute for Oncology and Radiology of Serbia, BelgradeSerbia; 3 Department of Medical Oncology, Institute for Oncology and Radiology of Serbia, BelgradeSerbia; 4 Department of Radiology, Institute for Oncology and Radiology of Serbia, BelgradeSerbia

**Keywords:** Alpha-1-antitrypsin, polymorphism, lymphomas

## Abstract

Alpha-1-antitrypsin (AAT) or serine protease inhibitor A1 (SERPINA1) is an important serine protease inhibitor in humans. The main physiological role of AAT is to inhibit neutrophil elastase (NE) released from triggered neutrophils, with an additional lesser role in the defense against damage inflicted by other serine proteases, such as cathepsin G and proteinase 3. Although there is a reported association between AAT polymorphism and different types of cancer, this association with hematological malignancies (HM) is, as yet, unknown. We identified AAT phenotypes by isoelectric focusing (in the pH 4.2-4.9 range) in 151 serum samples from patients with HM (Hodgkins lymphomas, non-Hodgkins lymphomas and malignant monoclonal gammopathies). Healthy blood-donors constituted the control group (n = 272). The evaluated population of patients as well as the control group, were at Hardy-Weinberg equilibrium for the AAT gene (χ^2^ = 4.42, d.f.11, p = 0.96 and χ^2^ = 4.71, d.f.11, p = 0.97, respectively). There was no difference in the frequency of deficient AAT alleles (Pi Z and Pi S) between patients and control. However, we found a significantly higher frequency of PiM1M1 homozygote and PiM1 allele in HM patients than in control (for phenotype: f = 0.5166 and 0.4118 respectively, p = 0.037; for allele: f = 0.7020 and 0.6360 respectively, p = 0.05). In addition, PiM homozygotes in HM-patients were more numerous than in controls (59% and 48%, respectively, p = 0.044). PiM1 alleles and PiM1 homozygotes are both associated with hematological malignancies, although this is considered a functionally normal AAT variant.

Hematological malignancies are clinically and biochemically diverse disorders of unknown etiology and characterized by the disproportionate proliferation of one clone of B and/or T cells.

Alpha-1-antitrypsin (AAT) is a highly polymorphic plasma glycoprotein (53 kD), synthesized in hepatocytes (Koj *et al.*, 1978) and subsequently secreted into the plasma. It is also produced in smaller quantities by alveolar macrophages, circulating monocytes (Mornex *et al.*, 1986) and in lung-derived epithelial cells (Cichy *et al.*, 1997). AAT, also called serine proteinase inhibitor A1 (SERPINA1), is the archetypal extracellular serpin (SER*ine* P*roteinase* IN*hibitor*).

The target proteinases of AAT originate from the azurophilic granules of polymorphonuclear neutrophils, which, in turn, contain three serine proteinases, elastase, cathepsin G and proteinase 3. These proteinases participate in lysosomal bacterial digestion and neutrophil migration through the extracellular matrix at the sites of inflammation. The main physiological role of AAT is to inhibit neutrophil elastase (NE) in the lower respiratory tract, so as to protect the connective tissue against NE released from triggered neutrophils (Travis and Salvesen, 1983), with a lesser role in defending against damage by other serine proteinases, such as cathepsin G (Duranton *et al.*, 1998) and proteinase 3 (Rao *et al.*, 1991).

The AAT protein is encoded by the protease inhibitor (Pi) locus on chromosome 14q31-32.3. Through the technique of isoelectric focusing, about 100 genetic variants of AAT have been identified to date.

The most common alleles are the M variants, which are subdivided into six M subtypes, homozygous or heterozygous for the M1, M2, M3 and M4 alleles. When M variants are inherited in homozygous or heterozygous form, AAT serum levels are supposed to be normal (Brantly *et al.*, 1988). The common variants that lead to AAT plasma deficiency (AATD) are Z and S, which could result in early-onset chronic obstructive pulmonary diseases, these including emphysema and chronic bronchitis, as well as liver disease, expressed as neonatal cholestasis, that may give rise to juvenile cirrhosis or a slowly progressive liver disease in adults. Thus, individuals who are homozygous for AATD alleles can develop liver or lung diseases. Nevertheless, AATD heterozygotes, which have inherited one normal allele, have sufficient amounts of AAT and are therefore less prone to diseases.

Furthermore, AAT polymorphism was also investigated in various malignant diseases. It has been documented that AAT deficiency is associated with the increased risk involved with several types of cancer, namely lung cancer (Yang *et al.*, 1999, 2008; Topic *et al.*, 2006), liver cancer (Propst *et al.*, 1994), bladder cancer (Benkmann *et al.*, 1987), colorectal cancer (Yang *et al.*, 2000), and gall bladder adenocarcinoma (Callea *et al.*, 1982b). To our knowledge, there are only a few studies which have investigated the association of AAT polymorphism with hematological malignancies. In studies by Ananthakrishanan *et al.* (1979) and Callea *et al.* (1982a), the, increased incidence of deficient Z and S alleles among patients with paraproteinemias and malignant lymphoma was evident. Therefore, the exact role of AAT variants as a risk factor in hematological malignancies remains unknown. Our intention was to investigate AAT polymorphism in patients with hematological malignancies, in particular lymphomas.

In order to evaluate the distribution of Pi phenotypes and PiM subtypes, we performed Pi phenotyping on healthy blood donors and patients with Hodgkins lymphomas, non-Hodgkins lymphomas and malignant monoclonal gammopathies. The patient group consisted of 151 patients (119 males and 32 females, aged between 21 and 69), with Hodgkins lymphomas (HL, n = 26), non-Hodgkins lymphomas (NHL, n = 35) and malignant monoclonal gammopathies (MMG, n = 90), and which had been admitted to the Institute of Oncology and Radiology of Serbia and the Military Medical Academy, Belgrade.

Diagnosis and typisation of malignant lymphomas was made by histological examination of biopsied material stained by HE and/or the MGG technique, with additional immunohistochemical determination of at least LCA, CD3, CD5, CD10, CD20, CD23 and CD45. Additional immunohistochemistry was carried out as required. Malignant monoclonal gammopathies were revealed with monoclonal immunoglobulin or Bence Jones protein in serum and/or urine by electrophoresis and immunofixation.

The control group consisted of 272 healthy blood-donors without any type of malignancies (221 males and 51 females, aged 20-65). Investigated populations, both control and patents, were proportional to the ethnic background.

The protocol was approved by local research ethics committees, and informed consent was obtained from all the participants.

Pi phenotyping of serum samples was carried out by isoelectric focusing (pH range 4.2-4.9) according to the method by Kishimoto *et al.* (1990). By using such a narrow range of pH for gradient and self-casted 0.2 mm thin polyacrylamide gels, we could clearly distinguish the three M subtypes (M1, M2 and M3). Before focusing serum, samples were pretreated by dithioerythritol.

The χ^2^ test was used to assess whether control and patient groups were in Hardy-Weinberg equilibrium for the AAT gene. Investigation of differences in the frequencies of AAT phenotypes and alleles, as well as the frequency of M homozygote and M heterozygote between patients and controls, were investigated, also by using the χ^2^ test (2 x 2 contingency table). The Fisher exact test was used when n < 5. P values of < 0.05 were considered significant. For statistical analysis, we used STATISTICA 6.0^®^*software.*

There was no deviation from Hardy-Weinberg equilibrium in either study group (χ^2^ = 4.71, d.f.11, p = 0.97 for control; χ^2^ = 4.42, d.f.11, p = 0.96 for HM patients).

The differences in distribution of Pi phenotypes and gene frequencies between patients and control are shown in Table 1. The M1 homozygote was significantly more frequent in HM patients than in controls (f = 0.5166 and f = 0.4118 respectively, p = 0.037). In addition, the M1 allele was more frequent in patients than in control group (f = 0.7020 and 0.6360 respectively, p = 0.05). Furthermore, we evaluated differences in distribution among the M subtypes, the homozygotes (M1M1, M2M2, and M3M3) and heterozygotes (M1M2, M1M3 and M2M3). This revealed there were more M homozygotes in patients than in the control group (59% and 48%, respectively, p = 0.044).

Although PiM subtypes are not linked to any disease, there are some studies in which a distinct association between a lowered M3 allele and monoclonal gammopathies and acute myeloid leukemia has been observed (Jelic *et al.*,1996; Janardhana and Propert*,* 1990).

It is generally accepted that AATD phenotypes are clinically important due to a firm linkage with liver and lung diseases. Several mutations of AAT are associated with low plasma level of AAT and the most common are Z and S variants. To date, few studies have been dedicated to examining the correlation of AAT polymorphism with hematological malignancies, although two such studies have described the association between the PiMZ phenotype and p*araproteinemias* and lymphomas (Ananthakrishanan *et al.*, 1979; Callea *et al.*,1982a). These authors presented the hypothesis that AAT could be included in the development of immunopathological disorders. In our study, no differences were apparent in the frequency of AATD phenotypes (MZ and MS) or alleles (Z and S) between HM patients and healthy individuals. Our results are in accordance with those gathered by El-Akawi *et al.* (2008) which showed that in all breast cancer patients, the normal allele (PiMM) was homozygous, though this is not the case for PiZ or PiS. Nevertheless, a borderline difference between patients and control in the distribution of PiM subtypes was manifest. There were more PiM1M1 homozygotes and M1 alleles in patients than in control individuals. Furthermore, we discovered higher PiM homozygote (M1M1, M2M2 and M3M3) frequency in patients than in controls (59% and 48% respectively, p = 0.044). Thus, PiM heterozygotes (M1M2, M1M3 and M2M3) were more abundant in healthy individuals than in patients (52% and 41% respectively, p = 0.044).

The mechanism that leads to an increase in PiM homozygosity in hematological malignancies is unknown. We believe that enlightenment on this will contribute to a better understanding of HM pathogenesis. Although it is considered that PiM subtypes are not related to diseases, we will present a hypothesis regarding their connection with HM.

The discrepancy regarding PiM homozygosity between patients and control could be due to certain advantages arising from PiM heterozygotes in healthy individuals. Two articles have been published which reported quantitative differences between PiM homozygotes and PiM heterozygotes. From the Beckman study (Beckman and Beckman, 1980), it was shown that in PiM homozygotes the AAT serum level was lower than in PiM heterozygotes. In the study by Oakeshott *et al.*,(1985), the functional activity of AAT was defined in a large sample group of blood donors, this including serum elastase inhibitory capacity. In their study it appeared that the values for elastase inhibitory capacity, concentration of AAT and their ratio (ratio = EIC/AAT) in PiM homozygotes were lower than in PiM heterozygotes. According to Gibson *et al.* (1983), it was shown that the lung function in females was greater in PiM heterozygotes than in PiM homozygotes or in any other Pi phenotypes with low α_1_-antitrypsin activity. Based on these studies, we assumed that increased PiM homozygote frequency in patients with HM may be due to their slightly reduced concentration and functional activity, which could lead to perturbation of the protease-antiprotease balance. In fact, imbalance between neutrophil elastase and alpha-1-antitrypsin is generally considered to be the cause of tissue damage, thereby creating a favorable tissue environment for carcinogens and tumor progression (Sun and Yang, 2004). Having in mind that PiM homozygotes with hematological malignancies are not really AAT deficient, as is the case of PiZ homozygotes, we can assume that an unknown risk factor (or factors) may be involved in the development of protease-antiprotease imbalance.

To conclude, the higher frequency of PiM1 homozygotes and PiM1 allele in patients with hematological malignancies could be a consequence of a so far unknown association between the main serine protease inhibitor and hematological malignancies.

## Figures and Tables

**Table 1:** Comparison of Pi phenotypes and allele frequencies between 151 HM patients and 272 healthy individuals (relative frequency/number).

Phenotype	Patients	Control	p*	Allele	Patients	Control	p**
M1	0.5166/78	0.4118/112	0.037	M1	0.7020/212	0.6360/346	0.050
M2	0.0464/7	0.0404/11	0.772	M2	0.1755/53	0.2059/112	0.289
M3	0.0132/2	0.0074/2	0.548	M3	0.1126/34	0.1324/72	0.405
M1M2	0.2053/31	0.2463/67	0.330	Z	0.0066/2	0.0165/9	0.222
M1M3	0.1457/22	0.1728/47	0.469	S	0.0033/1	0.0092/5	0.328
M2M3	0.0530/8	0.0699/19	0.496				
M1Z	0.0132/2	0.0221/6	0.505				
M2Z	0.0000/0	0.0074/2	0.290				
M3Z	0.0000/0	0.0037/1	0.455				
M1S	0.0066/1	0.0074/2	0.931				
M2S	0.0000/0	0.0074/2	0.290				
M3S	0.0000/0	0.0037/1	0.455				

*differences in frequencies of AAT phenotypes between patients and control. **differences in frequencies of AAT alleles between patients and control.
